# Predicting the Postmortem Interval Based on Gravesoil Microbiome Data and a Random Forest Model

**DOI:** 10.3390/microorganisms11010056

**Published:** 2022-12-24

**Authors:** Chunhong Cui, Yang Song, Dongmei Mao, Yajun Cao, Bowen Qiu, Peng Gui, Hui Wang, Xingchun Zhao, Zhi Huang, Liqiong Sun, Zengtao Zhong

**Affiliations:** 1 College of Life Sciences, Nanjing Agricultural University, Nanjing 210095, China; 2 College of Resource and Environment, Nanjing Agricultural University, Nanjing 210095, China; 3 Institute of Forensic Science, Ministry of Public Security, Beijing 100038, China; 4 Key Laboratory of Forensic Genetics of Ministry of Public Security, Beijing 100038, China; 5 College of Horticulture, Nanjing Agricultural University, Nanjing 210095, China

**Keywords:** postmortem interval, decomposition, bacterial community, machine learning algorithm

## Abstract

The estimation of a postmortem interval (PMI) is particularly important for forensic investigations. The aim of this study was to assess the succession of bacterial communities associated with the decomposition of mouse cadavers and determine the most important biomarker taxa for estimating PMIs. High-throughput sequencing was used to investigate the bacterial communities of gravesoil samples with different PMIs, and a random forest model was used to identify biomarker taxa. Redundancy analysis was used to determine the significance of environmental factors that were related to bacterial communities. Our data showed that the relative abundance of Proteobacteria, Bacteroidetes and Firmicutes showed an increasing trend during decomposition, but that of Acidobacteria, Actinobacteria and Chloroflexi decreased. At the genus level, *Pseudomonas* was the most abundant bacterial group, showing a trend similar to that of Proteobacteria. Soil temperature, total nitrogen, NH_4_^+^-N and NO_3_^−^-N levels were significantly related to the relative abundance of bacterial communities. Random forest models could predict PMIs with a mean absolute error of 1.27 days within 36 days of decomposition and identified 18 important biomarker taxa, such as *Sphingobacterium*, *Solirubrobacter* and *Pseudomonas*. Our results highlighted that microbiome data combined with machine learning algorithms could provide accurate models for predicting PMIs in forensic science and provide a better understanding of decomposition processes.

## 1. Introduction

The postmortem interval (PMI) is one of the most important aspects of forensic investigations because it provides necessary information in many criminal and legal cases [1,2]. Traditionally, estimation of the PMI has relied on evidence such as the physical processes that occur after death, including the drop in temperature of corpses, combined with livor mortis, rigor mortis and digestion of gastrointestinal contents [2,3]. However, the state of a dead body is difficult to maintain, and investigator experience surrounding environments and individual states usually affect the evaluation results. To avoid these shortcomings, some technologies have been applied to estimate the PMI, including biological chemistry [4,5,6], molecular biology [7,8], forensic entomology [9,10] and spectroscopic technology [11]. These methods can potentially provide significant information for PMI estimation. However, none of these methods are widely used by forensic investigators, and each of them faces some problems in practice. For example, the error range of forensic entomology can range from days to months when using this method to assess the PMI [12]. A study conducted by Pittner et al. [13] used a multidisciplinary approach to investigate postmortem changes, including morphology, skeletal muscle protein decomposition, presence of insects and other necrophilous animals and microbial communities, and summarized the current possibilities and limitations of these methods for PMI estimation. Currently, microbial approaches have started to draw more attention because forensically relevant microbial profiles could provide some evidence for PMI estimation or, at the very least, complement traditional investigative methods [14,15].

Microorganisms are abundant in almost all environments and take part in important ecological functions such as decomposition [16,17]. In the decomposition process, cadavers can be used as nutrients for microbial growth, and microbial communities respond rapidly to nutrient changes, showing a succession pattern [18]. Several studies have demonstrated that microbes can be used as a “clock” to estimate the PMI during cadaver decomposition [12,19], and these studies are associated with the skin [20,21], gut/intestine [22,23], oral cavity [24,25], gravesoil [26] and even bone [27]. In some studies, high-throughput sequencing and a variety of model-based statistical approaches, such as machine learning algorithms, were used to estimate the PMI. For example, based on 16S rRNA gene high-throughput sequencing data and random forest models, Zhang et al. [26] found that gravesoil, rectum and skin samples of buried cadavers could be used to predict the PMI, with a mean absolute error (MAE) of 1.82, 2.06 and 2.13 days within 60 days of decomposition, respectively. Another study used high-throughput sequencing to study the microbial communities of decomposing mouse cadavers, and the results showed that the MAE of the random forest model at 48 days was approximately 3 days [12].

Microbial succession after death is extremely complex and is affected by multiple environmental factors [28,29,30]. Currently, high-throughput sequencing technology and subsequent bioinformatics analysis can provide necessary information to gain insight into the complex microbial community composition in various environments [31]. Although postmortem microbiomes have been identified by previous studies, further research is still needed to obtain more information. In this study, the first objective was to determine the PMI based on bacterial community succession 36 days after the death of mice using high-throughput sequencing and random forest regression models and to determine bacterial biomarkers. Second, we aimed to identify the relationships between environmental factors and bacterial communities. This study helps us understand the relationship between the postmortem microbiome and decomposition and further provides theoretical evidence for forensic science and the criminal justice system.

## 2. Materials and Methods

### 2.1. Experimental Design and Sample Collection

All experiments were approved by the Animal Care and Use Committee of Nanjing Agricultural University (Nanjing, China) (permit number: SYXK (Su) 2017-0007). Sixty-five ICR mice (males, 20 ± 2 g) were acquired from Shanghai SLAC Laboratory Animal Co., Ltd (Shanghai, China). After one week of adaptive feeding, the mice were humanely euthanized by cervical dislocation. The experiment was conducted in a forest, in a small geographic range (Figure S1), where the soil was loose and the area was flat. Sixty-five cadavers were buried separately in 20 cm × 20 cm × 20 cm square graves (118°50′ E, 32°4′ N), and the distance between each pair of graves was greater than 10 cm. Each mouse was separately placed in a grave, and the soil was loosely placed back on top of the buried mouse. The soils under buried cadavers (depth ≤ 0.5 cm) were considered gravesoils. Five graves were excavated immediately, and after removing the mice, the gravesoils were carefully collected as controls (day 0). Then, five graves were randomly excavated every 3 days (days 3, 6, 9, 12, 15, 18, 21, 24, 27, 30, 33 and 36), and the gravesoils were collected accordingly. Each soil sample (approximately 3–5 g) was placed into a 10-mL sterile plastic tube and then immediately placed in a box filled with ice. After being taken back to the laboratory, each soil sample was divided into three parts. One part was stored at −80 °C for the extraction of soil DNA. Another part was used to determine ammonium (NH_4_^+^-N) and nitrate (NO_3_^−^-N) contents. The third part was air-dried in the laboratory for soil chemical analysis.

### 2.2. Soil Physical and Chemical Properties

The soil temperature and humidity of each grave were measured using a thermohygrometer (TA8672, TASI, Suzhou, China). Soil pH was determined using a pH meter (PB-10, Sartorius, Germany) in a 1:5 soil/water mixture. Total organic carbon (TOC) and total nitrogen (TN) contents were analyzed according to the methods of Bao [32]. For TOC, the soil sample was oxidized with K_2_Cr_2_O_7_-H_2_SO_4_ and titrated with a standard FeSO_4_ solution (phenanthroline indicator). For TN, the soil sample was catalyzed by an accelerator (K_2_SO_4_: CuSO_4_: Se (w: w: w) = 100: 10: 1) and heated using a boiling furnace. The final liquid was diluted with H_2_O and analyzed using a continuous flow analytical system (San^++^ System, Skalar, Holland). NH_4_^+^-N and NO_3_^−^-N were extracted at a ratio of 1 g of fresh soil to 10 mL of 2 M KCl for 1 h. Then, the liquid was analyzed using the aforementioned continuous flow analytical system.

### 2.3. DNA Extraction, PCR Amplification and Sequencing

Genomic DNA of each soil sample was extracted from 0.5 g of gravesoil using a Fast DNA™ Spin Kit for Soil (MP Bio, Santa Ana, CA, USA) according to the instructions. The primer set 341F (5′-CCT AYG GGR BGC ASC AG-3′)/806R (5′- GGA CTA CNN GGG TAT CTA AT-3′) was selected to amplify bacterial 16S rRNA gene sequences. PCR was performed using a GeneAmp PCR System 9700 (ABI), and the reaction mixture (20 μL) comprised 4 μL of 5×FastPfu Buffer (TransStart, TransGen Biotech, Beijing, China), 1 μL (50 ng/μL) of template DNA solution, 2 μL of dNTP mixture (2.5 mmol/μL), 0.8 μL of each primer (5 μmol/μL), 0.4 μL of FastPfu polymerase and 11 μL of sterilized distilled water. The PCR cycling parameters were as follows: 95 °C for 5 min; 34 cycles of denaturation at 95 °C for 30 s, annealing at 57 °C for 30 s, and extension at 72 °C for 45 s; a final extension at 72 °C for 10 min. The PCR products were purified using an Axy Prep DNA Cell Extraction Kit (AXYGEN, Corning, America) following the instructions and then sequenced with an Illumina MiSeq FGX platform (Biozeron Co., Ltd, Shanghai, China.) according to the manufacturer’s protocols. The sequence files were submitted to the Genome Sequence Archive (https://ngdc.cncb.ac.cn/gsa/ accessed on 6 April 2022) under accession number CRA006561.

### 2.4. Data Analysis

High-throughput sequencing reads were analyzed using QIIME2 [33]. Briefly, the reads were filtered, denoised and merged, and chimeras were removed using DADA2 for quality control. Subsequently, mitochondria- and/or chloroplast-related sequences were removed based on the Greengenes database (version 13.8). From the reads, the amplicon sequence variants (ASVs) were clustered using the DADA2 function according to the SILVA database (version 132). In addition, the sequences were rarefied to the minimum number of bacterial sequences (*n* = 21,310 sequences). The alpha-diversity indices, including the Shannon and Chao 1 indices, were determined using QIIME2. Nonmetric multidimensional scaling (NMDS) was used to determine the clustering of different soil samples based on the Bray–Curtis distance using R package vegan. PERMANOVA was used to examine the difference in bacterial community compositions among samples (R software (version 4.02), vegan). Redundancy analysis (RDA) was performed to arrange bacterial communities based on environmental factors. One-way ANOVA with the Student–Newman–Keuls (SNK) test was used to compare the differences among samples.

A random forest (RF) model, a machine learning method, was used in this study to generate PMI prediction models based on the relative abundances of bacterial taxa against the actual PMI (R package ‘randomForest’). Bacterial taxa were tacitly ranked in the RF model (feature importance) by 100 iterations. The number of biomarkers was determined by 10-fold cross-validation implemented with the rfcv() function. The minimum cross-validation error was obtained accordingly. In addition, a RF regression model based on feature species was established to predict the PMI. The MAE and goodness-of-fit (R^2^) were used to measure the accuracy and efficiency of the models. The MAE was calculated according to the methods of Zhang et al. [26] and Liu et al. [34].

## 3. Results

### 3.1. Sequencing and Bacterial Community Composition during Decomposition

High-throughput sequencing yielded a total of 2146,876 high-quality sequences, and 21,310–53,446 reads were obtained from each sample. In total, 1362 ASVs were detected in all soil samples. The number of ASVs and the Shannon index were selected to estimate the bacterial richness and diversity during cadaver decomposition. As shown in Figure S2, the Shannon indices generally showed no significant differences among the first 24 days (*p* > 0.05). By comparison, an obviously decreasing trend was observed in the later stage (days 27 to 36) of decomposition (*p* < 0.05). A similar pattern was observed regarding the number of ASVs, except on the 27th day.

**Figure 1:**
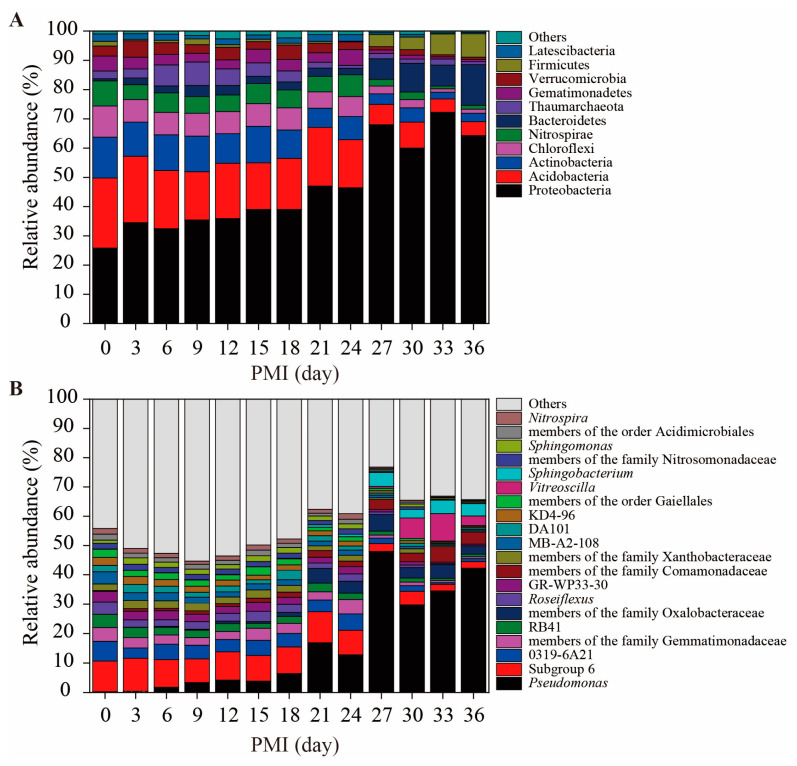
The relative abundances of the main bacterial members for different gravesoils. The stacked bar graph represents the relative abundances (%) of the major bacterial community, and only the average relative abundance > 1% for phylum (**A**) and the top genera (**B**) for genus across all the samples are shown. The relative abundance of each taxon is the average value of five replications (four replications for days 3, 27 and 30).

There were 11 dominant bacterial phyla (mean relative abundance > 1%) across all PMI-related soil samples: Proteobacteria, Acidobacteria, Actinobacteria, Chloroflexi, Nitrospirae, Bacteroidetes, Thaumarchaeota, Gemmatimonadetes, Verrucomicrobia, Firmicutes and Latescibacteria (Figure 1A). As shown in Figure 1A, the samples exhibited differences in the relative abundance of each bacterial group. Notably, the relative abundances of Proteobacteria and Bacteroidetes generally increased during cadaver decomposition. In contrast, the relative abundances of Acidobacteria, Actinobacteria, Chloroflexi and Nitrospirae showed opposite patterns. Furthermore, the top 20 genera across all samples are illustrated in Figure 1B, showing that *Pseudomonas*, subgroup 6 and 18 other bacterial groups were the dominant genera. Similarly, the relative abundances of *Pseudomonas*, members of the family Oxalobacteraceae, members of the family Comamonadaceae, *Vitreoscilla* and *Sphingobacterium* increased with an increasing PMI, and the relative abundances of subgroup 6, 0319-6A21, members of the family Gemmatimonadaceae, RB41 (subgroup 4), *Roseiflexus*, GR-WP33-30, members of the family Xanthobacteraceae and MB-A2-108 decreased.

### 3.2. Bacterial Succession Pattern during Cadaver Decomposition

**Figure 2:**
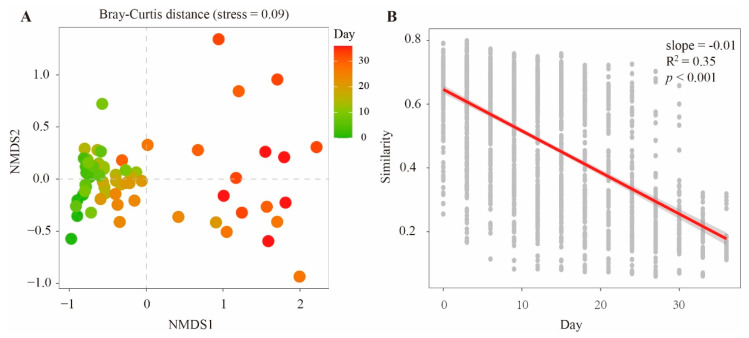
Bacterial communities changed with the PMI. (**A**) Nonmetric multidimensional scaling (NMDS) plots of bacterial communities based on the Bray−Curtis distances. (**B**) Significant linear relationships between the similarities of bacterial communities and the number of days of decomposition were observed in gravesoil.

To compare the dissimilarity of bacterial communities during cadaver decomposition, NMDS based on the Bray–Curtis distance was used to display the distribution of all soil samples in a two-dimensional space (Figure 2A). The results showed that the samples with different PMIs were separated from each other (F. model = 5.25, R^2^ = 0.563, *p* < 0.001). PERMANOVA based on the Bray–Curtis distance supported the dissimilarities of bacterial communities between most pairs of PMI-related samples (Table S1), suggesting that the bacterial communities changed along with the PMI. Furthermore, a linear model was analyzed to determine relationships between similarities of bacterial communities and PMIs. A negative slope (slope = −0.01, R^2^ = 0.35, *p* < 0.05) was observed based on the plots of bacterial community similarity versus PMI (Figure 2B). The curve suggested a succession pattern of microbial communities and consequently estimated the PMI according to the microbial community composition.

### 3.3. Effect of Environmental Factors on the Bacterial Community Composition

**Figure 3:**
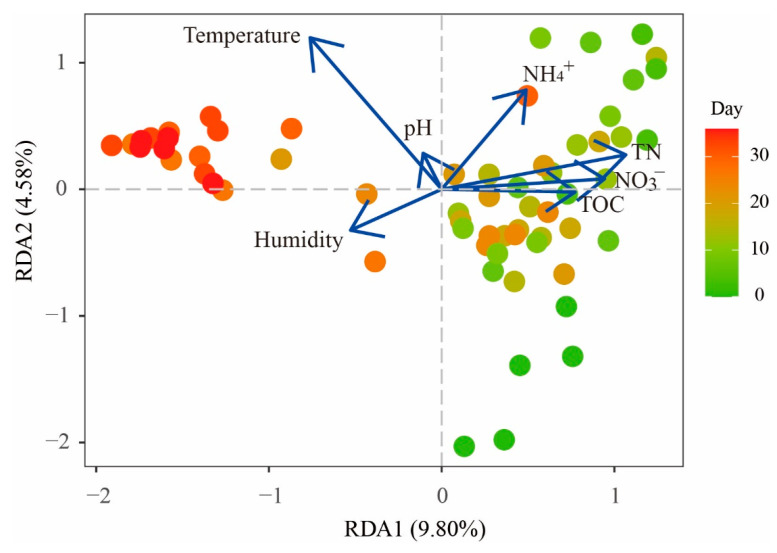
RDA ordination diagram of environmental factors in relation to soil samples. Environmental factors are indicated by lines with arrows, and the soil samples are represented by dots.

RDA was performed to determine the most significant environmental factors shaping bacterial communities during cadaver decomposition (Figure 3). The data indicated that environmental factors were significantly related to the bacterial community composition. The first two axes together explained 14.38% of the total variation in the bacterial communities. The RDA results showed that the bacterial communities were significantly impacted by TN, NH_4_^+^-N and NO_3_^−^-N levels and temperature (Table 1). Among them, temperature had the greatest impact, explaining 6.35% of the explained variation in the dataset, followed by TN (5.62%), NO_3_^−^-N (3.06%) and NH_4_^+^-N (2.44%) levels.

### 3.4. Bacterial Taxonomic Biomarkers for the PMI Determined Using the RF Model

**Figure 4:**
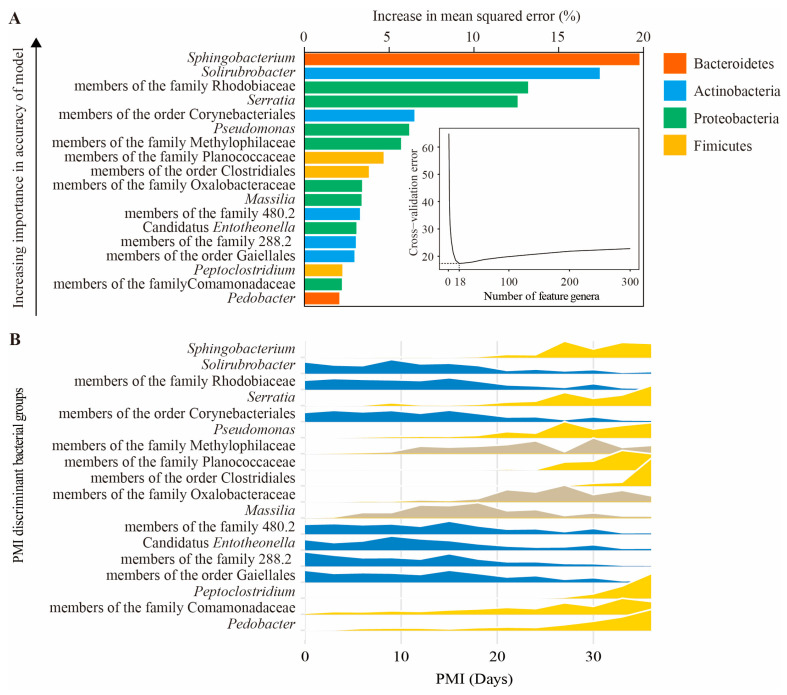
Bacterial taxonomic biomarkers of gravesoils during cadaver decomposition. (**A**) The top 18 biomarker bacterial genera were identified by applying a random forest model of their relative abundances in gravesoils against PMIs. Biomarker taxa are ranked in descending order of importance to the accuracy of the model. The inset represents a 10-fold cross-validation error as a function of the number of input genera used to regress against PMIs. (**B**) Abundance profiles for PMI-discriminant genera in gravesoils. Genera are colored by their classification as early, late, or complex colonizer patterns. There were 8 genera (yellow) with increasing relative abundances, 3 genera (gray) with complex patterns and 7 genera (blue) with decreasing relative abundances during cadaver decomposition.

To process the large datasets obtained by high-throughput sequencing, we regressed the relative abundance of soil bacterial communities at the genus level against the PMI using the RF machine learning algorithm. The model explained 85.7% of the bacterial community composition variance related to the PMI. We performed 10-fold cross-validation to reveal the importance of bacterial genera as biomarker taxa during cadaver decomposition. The minimum cross-validation error was obtained using 18 important genera. The top 18 bacterial groups at the genus level across PMIs are shown in Figure 4A in the order of time-discriminatory importance. *Sphingobacterium* was the most important genus in the process of cadaver decomposition, followed by *Solirubrobacter*, members of the family Rhodobiaceae and *Serratia*. As shown in Figure 4B, some biomarker taxa, such as *Solirubrobacter* and members of the family Rhodobiaceae, showed higher relative abundances in the early stage of decomposition, whereas *Sphingobacterium*, *Serratia* and *Pseudomonas* were abundant in the later stage of decomposition. A new RF model was established to regress the 18 biomarker taxa against the PMI, and the results showed that the new model could explain 83.9% of the variance.

**Figure 5:**
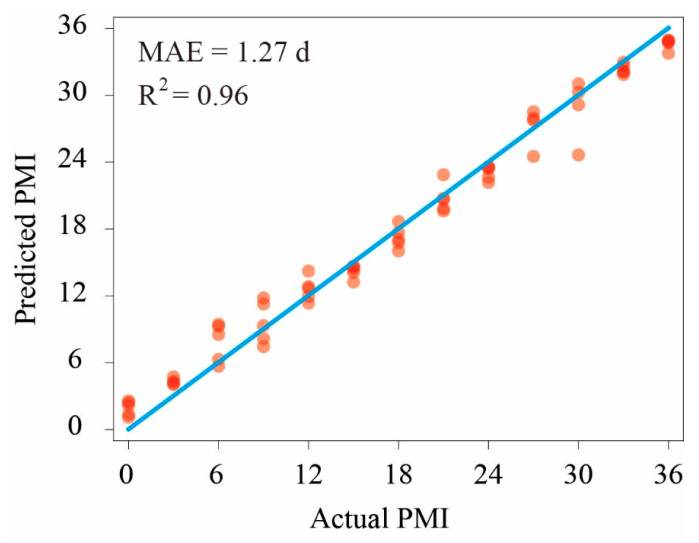
Random forest regression model. Each red dot represents a gravesoil sample. The blue straight line represents the predicted value, and the actual values are equal.

To accurately evaluate the prediction effect, the differences between the predicted and actual PMIs of each sample were analyzed using a RF regression model. The dataset was not divided into a test set and a training set because the number of soil samples was only 62. As shown in Figure 5, the R^2^ value was 0.96, which suggests that the regression effect of the RF algorithm was good. The MAE was 1.27 ± 0.18 d within 36 d of the decomposition process.

## 4. Discussion

Previous studies have demonstrated that microbiological methods can be used as promising tools to predict postmortem changes in forensic investigations [14,18,26]. Based on the data of high-throughput sequencing and machine learning algorithms, PMIs have been estimated under different environmental conditions [21,35,36]. However, the relationship between buried cadavers and their related microbial community needs to be further studied. In this study, based on 16S-amplicon sequencing and the RF model, PMIs could be predicted with high accuracy using the succession of bacterial biomarkers.

Microorganisms are the most abundant and vital components of soils and are sensitive to environmental changes. During cadaver decomposition, nutrient-rich fluids are released into the underlying soil, which can greatly impact the composition of the nearby microbial community [37]. In this study, a similar pattern was also observed (Figure 1), showing the changes in bacterial community composition during cadaver decomposition. Generally, Proteobacteria, Acidobacteria, Actinobacteria and Chloroflexi were the dominant bacterial groups in the soil samples. These phyla are commonly found in forest soils [38]. Our data indicated that Proteobacteria was the most abundant bacterial phylum (25.8%−72.2% of the overall community) across all samples and increased in abundance during cadaver degradation. Bacteria within Proteobacteria are ubiquitous in various soil environments [39,40], and they are typical r-strategy bacteria that are generally considered fast-growing bacteria often connected with labile carbon sources [41,42,43,44]. In this study, we hypothesized that the degradation of cadavers increased the available nutrient levels in gravesoils and therefore resulted in the rapid enrichment of Proteobacteria. At the genus level, *Pseudomonas*, a member of gamma-Proteobacteria, was the most abundant group, and increased abundance of this genus was positively related to Proteobacteria (Figure 2). Moreover, Pearson correlation analysis suggested that the relative abundance of *Pseudomonas* was positively related to the concentration of NH_4_^+^-N (r = 0.45, *p* < 0.05). Previous studies have noted that members of *Pseudomonas* are versatile and involved in organic pollutant degradation [45], plant growth promotion [46], nitrification and denitrification [47,48]. In this study, the contents of NH_4_^+^-N in gravesoils gradually accumulated as cadavers decomposed (Figure S2). The large amount of ammonium provided enough substrate for nitrification and subsequent denitrification. Therefore, *Pseudomonas*, as a heterotrophic nitrifier and/or denitrifier, exhibited rapid growth at the later stage of cadaver decomposition.

Compared with that of Proteobacteria, the relative abundances of Acidobacteria, Actinobacteria and Chloroflexi changed with different patterns, showing decreasing trends at the later stage of decomposition (Figure 2). Acidobacteria are frequently reported to show a close correlation with soil pH [49,50]. Currently, this phylum has 26 accepted subdivisions [51]. However, not all of the subdivisions consistently favor low-pH conditions in soils [52]. For example, Gp6 could be either positively or negatively correlated with soil pH [53,54], indicating that the growth of Gp6 was not affected by pH. Notably, the relative abundance of Acidobacteria was negatively related to the concentration of NH_4_^+^-N (r = −0.63, *p* < 0.01, Table S2). This result was supported by Zhou et al. [55], who investigated the effects of inorganic nitrogen on rhizosphere bacterial communities of the tropical seagrass *Thalassia hemperichii* and found that the relative abundance of Acidobacteria decreased under ammonium enrichment treatment. Similarly, Liu et al. [56] found that the relative abundance of Acidobacteria decreased with an increasing NH_4_^+^-N dose. Members of Actinobacteria in general have shown an ability to adapt to resource-limited environments [41,57]. Ryckeboer et al. [58] highlighted that, compared with other microorganisms, Actinobacteria showed low competitiveness under high-nutrient conditions. Conversely, Actinobacteria and Acidobacteria were in the K-strategy groups [42,59]. According to these results, we suppose that cadaver burial resulted in greater microbial biomass, but not the absolute abundance of Acidobacteria and Actinobacteria. Therefore, their relative abundance decreased at the late stage of decomposition. The phylum Chloroflexi showed similar and negative responses to higher concentrations of NH_4_^+^-N. Some studies have indicated that additional N suppresses this phylum. For example, Fierer et al. [60] found that the relative abundance of Chloroflexi decreased when additional N was used. A study conducted by Eo and Park [61] reported a similar result and summarized that the suppressive effect of additional N was related to altered chemical properties (e.g., soil pH and available nutrients) and microbial interactions such as competition and antagonism.

In addition to those of the four aforementioned phyla, the relative abundances of Bacteroidetes and Firmicutes significantly increased from the 27th day. This result was supported by Procopio et al. [62], who investigated changes in soil microbial communities associated with the decomposition of buried carcasses and found that the relative abundances of Proteobacteria, Firmicutes and Bacteroidetes increased with increasing PMIs. Many genera belonging to Firmicutes were confirmed to have the ability to transform organic nitrogen to NH_4_^+^-N [63]. Our data showed that the relative abundance of Firmicutes was significantly correlated with the content of NH_4_^+^-N (r = 0.73, *p* < 0.01, Table S2), indicating that this phylum may play an important role in nitrogen cycling. In this study, *Sphingobacterium* and *Pedobacter*, affiliated with Sphingobacteriaceae, were the most abundant genera in the phylum Bacteroidetes and were also important biomarkers in the RF model (Figure 4). In a study conducted by Olakanye and Ralebitso-Senior [64], *Sphingobacterium* and *Pedobacter* were seasonal PMI markers for sandy clay soil. Nonetheless, more studies are still needed to understand the ecological roles of these genera.

The alpha diversity indices (number of ASVs and Shannon index) were higher at the early stage of decomposition than at the later stage, which was in accordance with the results of previous studies [26]. Cadaver decomposition resulted in enough vital resources for growth, and some r-strategy bacteria, such as *Pseudomonas*, grew quickly and finally became dominant groups. These dominant bacterial groups could be responsible for a “shield” effect due to a drastic evenness change, as all minor species become too scarce in terms of relative abundance to be detected by rarefied sequencing alone, therefore leading to an artificial apparent decrease in diversity.

Microbial communities in soils are sensitive to many environmental factors, such as pH, temperature, and nutrient content. In this study, RDA suggested that soil temperature and TN, NH_4_^+^-N and NO_3_^−^-N contents were the environmental factors that significantly affected the bacterial community composition (Figure 3 and Table 1). Some studies have revealed that temperature affects microbial succession. For example, in PMI estimation, scientists also confirmed the significant effects of temperature on microbial communities [19,28]. In this study, the experiment was done during a cold period of the year (Figure S2). As we know, soil enzymes produced by microorganisms are highly sensitive to temperature and biochemical reaction rates increased with temperature according to the Arrhenius law. Therefore, the decomposition process might be delayed by lower temperature, showing similar bacterial community composition from day 3 to day 18 (Figure 1A and Table S1). Compared with lower temperatures, microbial community composition usually showed rapid succession patterns within several days in the summer [22,34]. At the later stage of decomposition, bacterial community composition changed significantly, which might be due to the increased temperature and the gradual accumulation of nutrients. Our results also highlighted that soil nitrogen in different forms affected bacterial communities. One possible explanation was that a large amount of nitrogen (protein-, peptide-, amino- and NH_4_^+^-N) in gravesoil greatly impacted bacterial community composition during cadaver composition. Based on Figure S2, cadaver decomposition resulted in a sharp increase in NH_4_^+^-N content, particularly from the 21st day, and how NH_4_^+^-N affected the bacterial community composition is discussed above. However, due to the complexity of environmental factors, their effects on microbial communities during cadaver decomposition still need to be investigated.

Previous studies have demonstrated that machine learning methods are very powerful and ideal tools to calculate PMIs using complex microbiome data [20,21,26,34]. In this study, we combined bacterial community data and machine learning algorithms (RF model) to investigate microbial succession patterns during cadaver decomposition. Based on the RF model, 18 important bacterial genera were identified as biomarker taxa to explain the succession of bacterial communities during cadaver decomposition (Figure 4). The 18 biomarkers together could explain 83.9% of the variance, and the value was only slightly lower than that of the total microbiome data (85.7%). This result suggested that machine learning methods could simplify bacterial biomarkers that correlate with PMIs, supporting the hypothesis that machine learning methods could be applied to predict the PMI in forensic investigations. As shown in Figure 5, we found that the MAE during 36 days of decomposition was 1.27 ± 0.18 d. If the model is established, the PMI can be predicted based on real human microbiome data. Moreover, we found that the deviation between some predicted values and the actual values was high, which might be because of the small number of samples. Therefore, more samples should be collected for further research to minimize the deviation. In addition to the RF model, some other methods were also used to predict the PMI in previous studies, such as the *k*-nearest neighbor regressor, support vector machine and artificial neural network [20,34], and each of them has both strengths and weaknesses [21]. Therefore, multiple methods should be considered alone and/or in combination in future work to obtain more accurate PMIs.

## 5. Conclusions

In this study, a significant succession of bacterial communities was found during the 36-day cadaver decomposition. Bacterial communities were significantly related to temperature, TN, NH_4_^+^-N and NO_3_^−^-N. We used a machine learning algorithm to assess the microbiome data, and the results suggested that the RF model could effectively predict the PMI with an MAE of 1.27 ± 0.18 d during 36-day decomposition. We also observed several bacterial groups, such as *Sphingobacterium*, *Solirubrobacter*, members of the family Rhodobiaceae and *Pseudomonas*, that may facilitate the establishment of the PMI prediction model. Taken together, our data suggest that the combination of microbial methods and machine learning algorithms can support necessary information in forensic PMI investigations.

## Figures and Tables

**Table 1:** Correlation coefficients, r-squared and significance values for environmental factors with RDA axes.

	RDA1	RDA2	r^2^	*p* Value
pH	−0.130	0.991	0.018	0.56
TC	0.996	0.085	0.060	0.174
TN	0.899	0.438	0.143	0.012
NH_4_^+^	0.457	0.890	0.177	0.003
NO_3_^−^	0.969	0.246	0.095	0.05
Temperature	−0.303	0.953	0.345	0.001
Humidity	−0.729	−0.685	0.058	0.172

## Data Availability

Data is contained within the article or Supplementary Materials.

## Supplementary Materials

The following supporting information can be downloaded at https://www.mdpi.com/article/10.3390/microorganisms11010056/s1: Figure S1: The experimental site in this study; Figure S2: The changes in environmental factors in gravesoils during cadaver decomposition; Table S1: PERMANOVA (pairwise comparison) showing Bray–Curtis distance based on dissimilarity of bacterial communities among the soil samples; Table S2: Statistical analysis of correlation coefficients between the relative abundances of bacterial communities at the phylum level and environmental factors.

## References

[1] Saks M.J., Koehler J.J. (2005). The coming paradigm shift in forensic identification science. Science.

[2] Metcalf J.L. (2019). Estimating the postmortem interval using microbes: Knowledge gaps and a path to technology adoption. Forensic Sci. Int.-Gen..

[3] Henßge C., Madea B. (1996). Estimation of the time since death in the early post-mortem period. Forensic Sci. Int..

[4] Wyler D., Marty W., Bar W. (1994). Correlation between the post-mortem cell content of cerebrospinal fluid and time of death. Int. J. Legal Med..

[5] Mikami H., Terazawa K., Takatori T., Tokudome S., Tsukamoto T., Haga K. (1994). Estimation of time of death by quantification of melatonin in corpses. Int. J. Legal Med..

[6] Pittner S., Monticelli F.C., Pfisterer A., Zissler A., Sänger A.M., Stoiber W., Steinbacher P. (2016). Postmortem degradation of skeletal muscle proteins: A novel approach to determine the time since death. Int. J. Legal Med..

[7] Young S.T., Wells J.D., Hobbs G.R., Bishop C.P. (2013). Estimating postmortem interval using RNA degradation and morphological changes in tooth pulp. Forensic Sci. Int..

[8] Hansen J., Lesnikova I., Funder A.M., Banner J. (2014). DNA and RNA analysis of blood and muscle from bodies with variable postmortem intervals. Forensic Sci. Med. Pathol..

[9] Amendt J., Campobasso C.P., Gaudry E., Reiter C., LeBlanc H.N., Hall M.J.R. (2007). Best practice in forensic entomology—Standards and guidelines. Int. J. Legal Med..

[10] Guo Y., Cai J., Chang Y., Li X., Liu Q., Wang X., Wang X., Zhong M., Wen J., Wang J. (2011). Identification of forensically important sarcophagid flies (Diptera: Sarcophagidae) in China, based on COI and 16S rDNA gene sequences. J. Forensic Sci..

[11] Zhang J., Li B., Wang Q., Wei X., Feng W., Chen Y., Huang P., Wang Z. (2017). Application of fourier transform infrared spectroscopy with chemometrics on postmortem interval estimation based on pericardial fluids. Sci. Rep..

[12] Metcalf J.L., Parfrey L.W., Gonzalez A., Lauber C.L., Knight D., Ackermann G., Humohrey G.C., Gebert M.J., van Treuren W., Berg-Lyons D. (2013). A microbial clock provides an accurate estimate of the postmortem interval in a mouse model system. eLife.

[13] Pittner S., Bugelli V., Benbow M.E., Ehrenfellner B., Zissler A., Campobasso C.P., Oostra R.-J., Aalders M.C.G., Zehner R., Lutz L. (2020). The applicability of forensic time since death estimation methods for buried bodies in advanced decomposition stages. PLoS ONE.

[14] Robinson J.M., Pasternak Z., Mason C.E., Elhaik E. (2021). Forensic application of microbiomics: A review. Front. Microbiol..

[15] Phan K., Barash M., Spindler X., Gunn P., Roux C. (2020). Retrieving forensic information about the donor through bacterial profiling. Int. J. Legal Med..

[16] Lienhard P., Tivet F., Chabanne A., Dequiedt S., Lelièvre M., Sayphoummie S., Leudphanane B., Prévost-Bouré N.C., Séguy L., Maron P.-A. (2013). No-till and cover crops shift soil microbial abundance and diversity in Laos tropical grasslands. Agron. Sustain. Dev..

[17] Burcham Z.M., Pechal J.L., Schmidt C.J., Bose J.L., Rosch J.W., Benbow M.E., Jordan H.R. (2019). Bacterial community succession, transmigration, and differential gene transcription in a controlled vertebrate decomposition. Front. Microbiol..

[18] Pechal J.L., Crippen T.L., Benbow M.E., Tarone A.M., Dowd S., Tomberlin J.K. (2014). The potential use of bacterial community succession in forensics as described by high throughput metagenomic sequencing. Int. J. Legal Med..

[19] Carter D.O., Metcalf J.L., Bibat A., Knight R. (2015). Seasonal variation of postmortem microbial communities. Forensic Sci. Med. Pathol..

[20] Johnson H.R., Trinidad D.D., Guzman S., Khan Z., Parziale J.V., DeBruyn J.M., Lents N.H. (2016). A machine learning approach for using the postmortem skin microbiome to estimate the posymortem interval. PLoS ONE.

[21] Belk A., Xu Z.Z., Carter D.O., Lynne A., Bucheli S., Knight R., Metcalf J.L. (2018). Microbiome data accurately predicts the postmortem interval using random forest regression models. Genes.

[22] DeBruyn J.M., Hauther K.A. (2017). Postmortem succession of gut microbial communities in deceased human subjects. PeerJ.

[23] Liu R., Wang Q., Zhang K., Wu H., Wang G., Cai W., Yu K., Sun Q., Fan S., Wang Z. (2021). Analysis of postmortem intestinal microbiota successional patterns with application in postmortem interval estimation. Microb. Ecol..

[24] Adserias-Garriga J., Quijada N.M., Hernandez M., Lázaro D.R., Steadman D., Garcia-Gil L.J. (2017). Dynamics of the oral microbiota as a tool to estimate time since death. Mol. Oral Microbiol..

[25] Dong K., Xin Y., Cao F., Huang Z., Sun J., Peng M., Liu W., Shi P. (2019). Succession of oral microbiota community as a tool to estimate postmortem interval. Sci. Rep..

[26] Zhang J., Wang M., Qi X., Shi L., Zhang J., Zhang X., Yang T., Ren J., Liu F., Zhang G. (2021). Predicting the postmortem interval of burial cadavers based on microbial community succession. Forensic Sci. Int. Gen..

[27] Damann F.E., Williams D.E., Layton A.C. (2015). Potential use of bacterial community succession in decaying human bone for estimating postmortem interval. J. Forensic Sci..

[28] Carter D.O., Yellowlees D., Tibbett M. (2008). Temperature affects microbial decomposition of cadavers (*Rattus rattus*) in constrasting soils. Appl. Soil Ecol..

[29] Giles S.B., Harrison K., Errickson D., Márquez-Grant N. (2020). The effect of seasonality on the application of accumulated degree-days to estimate the early post-mortem interval. Forensic Sci. Int..

[30] Carter D.O., Yellowlees D., Tibbett M. (2010). Moisture can be the dominant environmental parameter governing cadaver decomposition in soil. Forensic Sci. Int..

[31] Huang Z., Zhao F., Wang M., Qi K., Wu J., Zhang S. (2019). Soil chemical properties and geographical distance exerted effects on arbuscular mycorrhizal fungal community composition in pear orchards in Jiangsu Province, China. Appl. Soil Ecol..

[32] Bao S.D. (2005). Agricultural Chemical Analysis of Soil.

[33] Bolyen E., Rideout J.R., Dillon M.R., Bokulich N.A., Abnet C.C., Al-Ghalith G.A., Alexander H., Alm E.J., Arumugam M., Asnicar F. (2019). Reproducible, interactive, scalable and extensible microbiome data science using QIIME2. Nat. Biotechnol..

[34] Liu R., Gu Y., Shen M., Li H., Zhang K., Wang Q., Wei X., Zhang H., Wu D., Yu K. (2020). Predicting postmortem interval based on microbial community sequences and machine learning algorithms. Environ. Microbiol..

[35] Hyun C., Kim H., Ryu S., Kim W. (2019). Preliminary study on microeukaryotic community analysis using NGS technology to determine postmortem submersion interval (PMSI) in the drowned pig. J. Microbiol..

[36] Metcalf J.L., Xu Z.Z., Weiss S., Lax S., van Treuren W., Hyde E.R., Sing S.J., Amir A., Larsen P., Sangwan N. (2016). Microbial community assembly and metabolic function during mammalian corpse decomposition. Science.

[37] Singh B., Minick K.J., Strickland M.S., Wicking K.G., Crippen T.L., Tarone A.M., Benbow M.E., Sufrin N., Tomberlin J.K., Pechal J.L. (2018). Temporal and spatial impact of human cadaver decomposition on soil bacterial and arthropod community structure and function. Front. Microbiol..

[38] Zhu K., Wang Q., Zhang Y., Zarif N., Ma S., Xu L. (2022). Variation in soil bacterial and fungal community composition at different successional stages of a broad-leaved Korean pine forest in the Lesser Hinggan Mountains. Forest.

[39] Neufeld J.D., Mohn W.W. (2005). Unexpectedly high bacterial diversity in arctic tundra relative to boreal forest soils, revealed by serial analysis of ribosomal sequence tags. Appl. Environ. Microbial..

[40] Curtis P., Nakatsu C.H., Konopka A. (2002). Aciduric proteobacteria isolated from pH 2.9 soil. Arch. Microbiol..

[41] Fierer N., Jackson R.B. (2006). The diversity and biogeography of soil bacterial communities. Proc. Natl. Acad. Sci. USA.

[42] Fierer N., Bradford M.A., Jackson R.B. (2007). Toward an ecological classification of soil bacteria. Ecology.

[43] Spain A.M., Krumholz L.R., Elshahed M.S. (2009). Abundance, composition, diversity and novelty of soil Proteobacteria. ISME J..

[44] Wang L., Li J., Yang F., E Y., Raza W., Huang Q., Shen Q. (2017). Application of bioorganic fertilizer significantly increased apple yield and shaped bacterial community structure in orchard soil. Microb. Ecol..

[45] Wasi S., Tabrez S., Ahmad M. (2013). Use of *Pseudomonas* spp. for the bioremediation of environmental pollutants: A review. Environ. Monit. Assess..

[46] Hol W.H.G., Bezemer M., Biere A. (2013). Getting the ecology into interactions between plants and the plant growth-promoting bacterium *Psedomonas fluorescens*. Front. Plant Sci..

[47] Zhang J., Wu P., Hao B., Yu Z. (2011). Heterotrophic nitrification and aerobic denitrification by the bacterium *Pseudomonas stutzeri* YZN-001. Bioresour. Technol..

[48] Zhang X., Xia Y., Zeng Y., Sun X., Tao R., Mei Y., Qu M. (2022). Simultaneous nitrification and denitrification by *Pseudomonas* sp. Y-5 in a high nitrogen environment. Environ. Sci. Pollut. Res..

[49] Navarrete A.A., Kuramae E.E., de Hollander M., Pijl A.S., van Veen J.A., Tsai S.M. (2013). Acidobacterial community responses to agricultural management of soybean in Amazon forest soils. FEMS Microbiol. Ecol..

[50] Foesel B.U., Nägele V., Naether A., Wüst P.K., Weinert J., Bonkowski M., Lohaus G., Polle A., Alt F., Oelmann Y. (2014). Determinants of *Acidobacteria* activity inferred from the relative abundances of 16S rRNA transcripts in German grassland and forest soils. Environ. Microbiol..

[51] Barns S.M., Takala S.L., Kuske C.R. (1999). Wide distribution and diversity of members of the bacterial kingdom *Acidobacterium* in the environment. Appl. Environ. Microbiol..

[52] Kielak A.M., Barreto C., Kowalchuk G.A., van Veen J.A., Kuramae E.E. (2016). The ecology of *Acidobacteria*: Moving beyond genes and genomes. Front. Microbiol..

[53] Chan O.C., Yang X., Fu Y., Feng Z., Sha L., Casper P., Zou X. (2006). 16S rRNA gene analyses of bacterial community structures in the soils of evergreen broad-leaved forests in south-west China. FEMS Microbiol. Ecol..

[54] Mukherjee S., Juottonen H., Siivonen P., Quesada C.L., Tuomi P., Pulkkinen P., Yrjälä K. (2014). Spatial patterns of microbial diversity and activity in an aged creosote-contaminated site. ISME J..

[55] Zhou W., Dong J., Ding D., Long L., Suo A., Lin X., Yang Q., Lin L., Zhang Y., Ling J. (2021). Rhizosphere microbiome dynamics in tropical seagrass under short-term inorganic nitrogen fertilization. Environ. Sci. Pollut. Res..

[56] Liu C., Dong Y., Hou L., Deng N., Jiao R. (2017). *Acidobacteria* community responses to nitrogen dose and form in Chinese fir plantations in southern China. Curr. Microbiol..

[57] Hartmann M., Lee S., Hallam S.J., Mohn W.W. (2009). Bacterial, archaeal and eukaryal community structures throughout soil horizons of harvested and naturally disturbed forest stands. Environ. Microbiol..

[58] Ryckeboer J., Mergaert J., Coosemans J., Deprins K., Swings J. (2003). Microbiological aspects of biowaste during composting in a monitored compost bin. J. Appl. Microbiol..

[59] Morrissey E.M., Mau R.L., Schwartz E., McHugh T.A., Dijkstra P., Koch B.J., Marks J.C., Hungate B.A. (2017). Bacterial carbon use plasticity, phylogenetic diversity and the priming of soil organic matter. ISME J..

[60] Fierer N., Lauber C.L., Ramirez K.S., Zaneveld J., Bradford M.A., Knight R. (2012). Comparative metagenomic phylogenetic and physiological analyses of soil microbial communities across nitrogen gradients. ISME J..

[61] Eo J., Park K.-C. (2016). Long-term effects of imbalanced fertilization on the composition and diversity of soil bacterial community. Agric. Ecosyst. Environ..

[62] Procopio N., Ghignone S., Williams A., Chamberlain A., Mello A., Buckley M. (2019). Metabarcoding to investigate changes in soil microbial communities within forensic burial contexts. Forensic Sci. Int.-Gen..

[63] Liu S., Li P., van Zwieten L., Tu J., Gan W., Lu S., Wang H., Wu L. (2021). Edaphic variables influence soil bacterial structure under successive fertilization of *Paulownia* plantation substituting native vegetation. J. Soil. Sediment..

[64] Olakanye A.O., Ralebitso-Senior T.K. (2018). Soil metabarcoding identifies season indicators and differentiators of pig and *Agrostis*/*Festuca* spp. decomposition. Forensic Sci. Int..

