# Supplementary material for: Predicting the Postmortem Interval Based on Gravesoil Microbiome Data and a Random Forest Model
Source: Microorganisms. 2022 Dec 24;11(1):56. doi: 10.3390/microorganisms11010056 (PMC9860995; doi:10.3390/microorganisms11010056)
Supplement: Supplementary file 1 [file microorganisms-11-00056-s001.zip › microorganisms-2033594-supplementary.pdf]

Supplementary Materials

Table S1. PERMANOVA (pairwise comparison) showing Bray−Curtis distance based on dissimilarity of bacterial communities among the soil samples.

Group	Sums of squares	Mean squares	F.Model	Variation (R^2)	Pr (>F)	Sig
day0/day12	0.214474	0.214474	4.561615	0.363139	0.01	**
day0/day15	0.175077	0.175077	3.785882	0.321222	0.005	**
day0/day18	0.188699	0.188699	4.418015	0.355775	0.007	**
day0/day21	0.328569	0.328569	5.171729	0.392639	0.004	**
day0/day24	0.268445	0.268445	4.474556	0.358695	0.009	**
day0/day27	0.906031	0.906031	8.759627	0.555827	0.01	**
day0/day30	0.754885	0.754885	8.039824	0.534569	0.003	**
day0/day33	1.258156	1.258156	11.06872	0.580465	0.008	**
day0/day36	1.439606	1.439606	21.50584	0.728867	0.011	*
day0/day3	0.160911	0.160911	4.222072	0.376229	0.015	*
day0/day6	0.145084	0.145084	2.893431	0.265612	0.015	*
day0/day9	0.178325	0.178325	2.855211	0.263027	0.007	**
day12/day15	0.065793	0.065793	1.30021	0.139804	0.135	NS
day12/day18	0.055971	0.055971	1.189143	0.129407	0.303	NS
day12/day21	0.153654	0.153654	2.263316	0.220525	0.013	*
day12/day24	0.168886	0.168886	2.62447	0.247021	0.007	**
day12/day27	0.736498	0.736498	6.793502	0.492515	0.008	**
day12/day30	0.473293	0.473293	4.786897	0.40612	0.018	*
day12/day33	0.96866	0.96866	8.207262	0.506394	0.008	**
day12/day36	1.163426	1.163426	16.31796	0.671025	0.009	**
day12/day3	0.100227	0.100227	2.325907	0.249403	0.028	*
day12/day6	0.075075	0.075075	1.377527	0.146897	0.071	NS
day12/day9	0.049924	0.049924	0.747213	0.085423	0.763	NS
day15/day18	0.051471	0.051471	1.111786	0.122016	0.314	NS
day15/day21	0.185826	0.185826	2.768707	0.257107	0.016	*
day15/day24	0.140361	0.140361	2.207684	0.216277	0.011	*
day15/day27	0.748866	0.748866	6.964283	0.498721	0.01	**
day15/day30	0.500599	0.500599	5.108672	0.421902	0.01	**
day15/day33	0.984773	0.984773	8.39874	0.512158	0.011	*
day15/day36	1.182285	1.182285	16.76406	0.676951	0.011	*
day15/day3	0.101727	0.101727	2.41008	0.256117	0.03	*
day15/day6	0.068303	0.068303	1.271291	0.137121	0.268	NS
day15/day9	0.055623	0.055623	0.842256	0.095254	0.557	NS
day18/day21	0.102358	0.102358	1.609837	0.16752	0.102	NS
day18/day24	0.098057	0.098057	1.633063	0.169527	0.018	*
day18/day27	0.653046	0.653046	6.310151	0.474086	0.004	**
day18/day30	0.418974	0.418974	4.459451	0.38915	0.02	*

day18/day33	0.877393	0.877393	7.715449	0.490947	0.013	*
day18/day36	1.05722	1.05722	15.7814	0.663603	0.011	*
day18/day3	0.107106	0.107106	2.805991	0.286151	0.016	*
day18/day6	0.07496	0.07496	1.493408	0.15731	0.04	*
day18/day9	0.080836	0.080836	1.29323	0.139158	0.171	NS
day21/day24	0.076562	0.076562	0.946781	0.105824	0.442	NS
day21/day27	0.384376	0.384376	3.019775	0.301382	0.056	NS
day21/day30	0.253983	0.253983	2.157031	0.23556	0.09	NS
day21/day33	0.560474	0.560474	4.165868	0.342423	0.006	**
day21/day36	0.69587	0.69587	7.924543	0.497631	0.009	**
day21/day3	0.216463	0.216463	3.493288	0.332907	0.011	*
day21/day6	0.208458	0.208458	2.935427	0.268433	0.008	**
day21/day9	0.211916	0.211916	2.543159	0.241214	0.011	*
day24/day27	0.481099	0.481099	3.903683	0.358015	0.032	*
day24/day30	0.3121	0.3121	2.744868	0.281673	0.036	*
day24/day33	0.687009	0.687009	5.244292	0.395966	0.005	**
day24/day36	0.825464	0.825464	9.795027	0.550436	0.009	**
day24/day3	0.226433	0.226433	3.909292	0.358345	0.015	*
day24/day6	0.204436	0.204436	3.029752	0.274689	0.009	**
day24/day9	0.202887	0.202887	2.542778	0.241187	0.007	**
day27/day30	0.156429	0.156429	0.902474	0.130746	0.538	NS
day27/day33	0.165888	0.165888	0.898711	0.113779	0.542	NS
day27/day36	0.122934	0.122934	0.93713	0.118069	0.505	NS
day27/day3	0.829036	0.829036	7.658151	0.560702	0.03	*
day27/day6	0.810564	0.810564	7.23819	0.508364	0.012	*
day27/day9	0.728118	0.728118	5.776114	0.452103	0.007	**
day30/day33	0.155261	0.155261	0.886978	0.112461	0.626	NS
day30/day36	0.193326	0.193326	1.589299	0.185032	0.163	NS
day30/day3	0.589751	0.589751	6.072017	0.502983	0.026	*
day30/day6	0.582238	0.582238	5.683423	0.448099	0.008	**
day30/day9	0.506667	0.506667	4.348425	0.383174	0.015	*
day33/day36	0.158147	0.158147	1.146429	0.125342	0.326	NS
day33/day3	1.070197	1.070197	8.973375	0.561771	0.012	*
day33/day6	1.076619	1.076619	8.88663	0.526252	0.007	**
day33/day9	0.964953	0.964953	7.230077	0.474724	0.011	*
day36/day3	1.246937	1.246937	18.93293	0.730073	0.01	**
day36/day6	1.273708	1.273708	17.11446	0.681458	0.007	**
day36/day9	1.15907	1.15907	13.36315	0.625523	0.009	**
day3/day6	0.056835	0.056835	1.21797	0.148208	0.231	NS
day3/day9	0.105025	0.105025	1.72921	0.198095	0.093	NS
day6/day9	0.060494	0.060494	0.864957	0.09757	0.629	NS

*, $p < 0.05$; **, $p < 0.01$; NS, $p > 0.05$

Table S2. Statistical analysis of correlation coefficients between the relative abundances of bacterial communities at the phylum level and environmental factors.

	**Temperature**	**Humidity**	**pH**	**NH_4^+-N**	**NO_3^--N**	**TOC**	**TN**
**Acidobacteria**	-0.59**	-0.16	-0.38**	-0.63**	0.32*	-0.09	-0.30*
**Actinobacteria**	-0.74**	-0.18	-0.49**	-0.70**	0.42**	-0.06	-0.34**
**Bacteroidetes**	0.50**	0.09	0.47**	0.48**	-0.21	0.21	0.47**
**Chloroflexi**	-0.71**	-0.13	-0.48**	-0.68**	0.27*	-0.17	-0.40**
**Firmicutes**	0.41**	0.21	0.45**	0.73**	-0.31*	0.01	0.31*
**Proteobacteria**	0.68**	0.22	0.43**	0.67**	-0.38**	0.04	0.26*

*, $p < 0.05$; **, $p < 0.01$

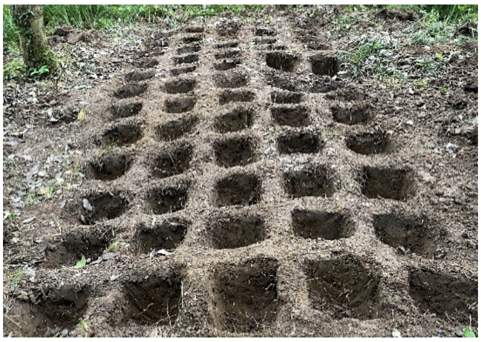

Figure S1. The experimental site in this study. The site is in a small geographic area, where is about 4 m long and 1.5 m width.

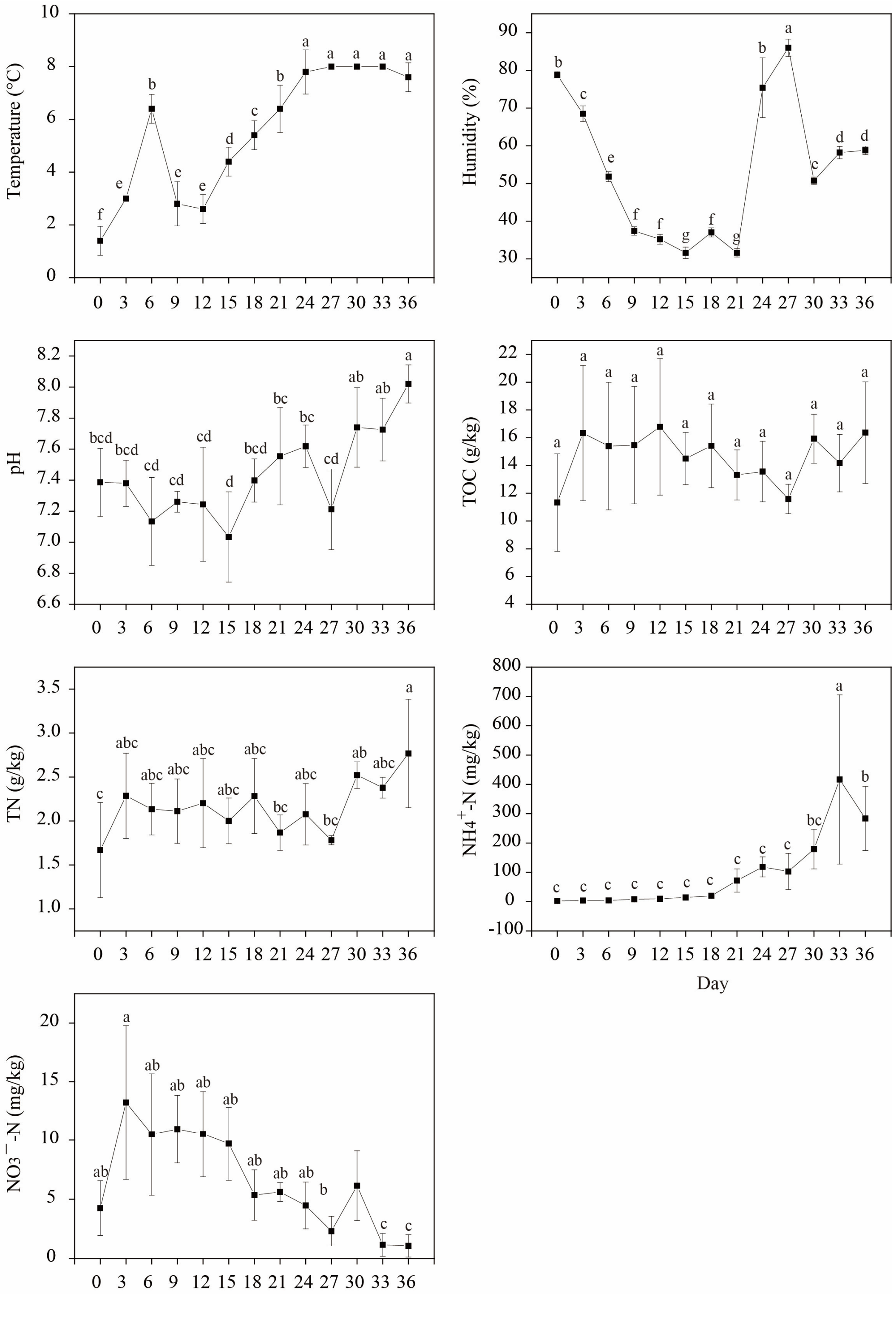

Figure S2. The changes in environmental factors in gravesoils during cadaver decomposition.
